# Dam Body Condition Score Alters Offspring Circulating Cortisol and Energy Metabolites in Holstein Calves but Did Not Affect Neonatal Leptin Surge

**DOI:** 10.3390/metabo13050631

**Published:** 2023-05-06

**Authors:** William E. Brown, Henry T. Holdorf, Sophia J. Kendall, Heather M. White

**Affiliations:** 1 Department of Animal and Dairy Sciences, University of Wisconsin-Madison, Madison, WI 53706, USA; 2 Department of Animal Sciences & Industry, Kansas State University, Manhattan, KS 66506, USA

**Keywords:** developmental programming, bovine, neonate

## Abstract

The neonatal leptin surge is important for hypothalamic development, feed intake regulation, and long-term metabolic control. In sheep, the leptin surge is eliminated with maternal overnutrition and an elevated dam body condition score (BCS), but this has not been assessed in dairy cattle. The aim of this study was to characterize the neonatal profile of leptin, cortisol and other key metabolites in calves born to Holstein cows with a range of BCS. Dam BCS was determined 21 d before expected parturition. Blood was collected from calves within 4 h of birth (d 0), and on days 1, 3, 5, and 7. Serum was analyzed for concentrations of leptin, cortisol, blood urea nitrogen, β-hydroxybutyrate (BHB), free fatty acids (FFA), triglycerides, and total protein (TP). Statistical analysis was performed separately for calves sired by Holstein (HOL) or Angus (HOL-ANG) bulls. Leptin tended to decrease after birth in HOL calves, but there was no evidence of an association between leptin and BCS. For HOL calves, the cortisol level increased with an increasing dam BCS on day 0 only. Dam BCS was variably associated with the calf BHB and TP levels, depending on the sire breed and day of age. Further investigation is required to elucidate the impacts of maternal dietary and energy status during gestation on offspring metabolism and performance, in addition to the potential impact of the absence of a leptin surge on long-term feed intake regulation in dairy cattle.

## 1. Introduction

Maternal nutrition during gestation can influence offspring performance through DNA methylation, placental efficiency, and fetal organ development [1,2]. Historically, in livestock sciences, research has evaluated the effects of maternal malnutrition during gestation on offspring performance because of the typical demands placed on the dam by environmental and production factors [1,2]. In contrast, the opposite may also present a challenge to both dam and offspring. A growing body of evidence demonstrates that maternal overnutrition during gestation has negative effects on the progeny’s ability to maintain a healthy body weight later in life in sheep [3,4] and rodents [5,6]. In dairy cattle, a greater gestational body condition score (BCS) predisposes the dam to excessive postpartum body fat mobilization and metabolic disorders [7], alters the neonate’s metabolic profile at birth [8], and is also positively associated with offspring BCS in adulthood [9].

A known modulator of lifelong feed intake and growth is leptin. In the neonate, leptin is critical for the innervation of arcuate nucleus fibers into the hypothalamus [10], with the hypothalamus serving as the site for central feed intake control. Occurring during the neonatal period, a characteristic leptin surge (LS) in mammals is known to modulate feed intake and body weight throughout life [11], and occurs between 5 to 10 d postnatally in sheep [12,13] and within 3 d postnatally in cattle [14,15]. While leptin release from adipose tissue in adult animals increases energy metabolism and decreases feed intake [16], leptin does not alter feed intake in mouse pups during the neonatal period [17]. In contrast, the experimental elimination of the neonatal LS via leptin antagonists leads to long-term leptin resistance and diet-induced obesity later in life [18]. Interestingly, the offspring of overweight ewes lack the neonatal LS [13], which may contribute to the association between dam gestational BCS, offspring BCS and feed intake perturbations. These perturbations may have multi-generational impacts, especially within breeding and production herds. In sheep, induced maternal obesity has a negative impact on the metabolism of adult daughters, and alters the metabolism and eliminates the LS of their granddaughters [19]. 

The potential for dairy cow BCS to have multigenerational impacts on health and metabolism warrants further investigation. The objective of this experiment was to characterize the neonatal profile of leptin, cortisol, and other serum metabolites (blood urea nitrogen, ß-hydroxybutyrate, free fatty acids, triglycerides, and total protein) in calves from normal or over-conditioned Holstein dams. We hypothesized that the offspring of dams with a greater BCS would have an ablated LS, elevated circulating cortisol levels at birth, and altered blood energy metabolites. 

## 2. Materials and Methods

### 2.1. Animal Use, Treatments, and Handling

All animal use and handling protocols for this study were approved by the University of Wisconsin—Madison College of Agricultural and Life Sciences Institutional Animal Care and Use Committee under protocol A006338. Multiparous Holstein cows (n = 94) were housed in a bedded pack facility 21 d before expected parturition and were offered one of four dietary treatments, which varied in their rumen-protected choline dose and formulation, as described previously [20]. The dietary treatments were not of primary interest in this study but served as an avenue via which to utilize an ongoing study aiming to test the hypothesis stated herein. The cows in this study were inseminated with either reverse-sorted Holstein semen to result in female calves (HOL), or conventional Angus semen (HOL-ANG). For the purposes of this study, only cows carrying female calves sired by either breed were enrolled; this was because previous research has demonstrated a difference between male and female bovine LS [15]. At enrollment, the cow body condition score (BCS) was evaluated by three trained investigators on a 5-point scale (1 = thin, 5 = fat; [21]). Cows with a BCS from 3.0 to 3.5 were classified as having a normal or ideal prepartum BCS [7], and cows with a BCS ≥ 3.75 were classified as having excessive prepartum BCS. The original project design sought to obtain complete and balanced datasets of Holstein- and Angus-sired calves from dams who had either normal or excessive BCS in a 2 × 2 factorial design. However, due to management and breeding decisions unrelated to this study, and likely typical of commercial dairies utilizing beef-on-dairy breeding strategies, only two HOL-ANG calves were born from dams with excessive BCS. Therefore, this combination of factors was eliminated from analysis because realistic comparisons could not be made. The final range of dam BCS was 3.0 to 4.68 for HOL and 3.83 to 4.58 for HOL-ANG. 

Upon birth, calves were separated from their dam and fed 3.8 L of previously frozen colostrum. Calves were individually housed in a straw-bedded hutch, and fed a traditional (0.8 kg dry matter/d; HOL-ANG) or accelerated (1.0 kg dry matter/d; HOL) milk replacer twice daily, and offered grain ad libitum. The complete calf nutrition and management procedures are outlined by Holdorf et al. [22].

### 2.2. Sample Collection and Analysis

Blood samples from calves were obtained via jugular venipuncture within 4 h of birth (d 0), and 4 h before afternoon feeding on postnatal days 1, 2, 3, 5 and 7. Blood was collected directly into a red-top vacuum tube containing a silica clotting activator (BD Vacutainer) and allowed to clot for at least 30 min. After sample coagulation, the tubes were centrifuged at 3000× *g* for 15 min. The resulting sera was aliquoted and immediately frozen at −80 °C for enzyme-linked immunoassay (ELISA) analysis or at −20 °C for other metabolite analysis.

Serum leptin was quantified using an internally validated competitive ELISA specific to bovine leptin (Cat. #CSB-E06771b; Cusabio Technology, LLC; Houston, TX, USA), according to the manufacturer’s instructions. Serum cortisol was quantified using an internally validated multi-species competitive ELISA (Cat. #K003-H1W/H5W; Arbor Assays; Ann Arbor, MI, USA) according to the manufacturer’s directions. For both assays, samples were analyzed in triplicate and an internal control sample was analyzed on each plate to assess repeatability across plates. The overall intraplate coefficients of variation (CV) were less than 4% and 6% for leptin and cortisol, respectively, and the inter-plate CV was less than 8% for both assays.

Other metabolites were assayed for samples obtained on days 0, 1, 2 and 5. The samples from days 3 and 7 were excluded from this analysis because a more comprehensive analysis of dam dietary affects have been presented previously [22]. The methods used to collect blood urea nitrogen (BUN) [23] and β-hydroxybutyrate have been reported previously [24] and were quantified according to those methods using the VETSPEC reagents on the Catachem Well-T Autoanalyzer (C264-04, BUN; C442-0A, BHB; Catachem; Oxford, CT, USA). Serum triglyceride concentrations were quantified on the CatachemWell-T Autoanalyzer using a modified protocol with the VETSPEC reagents (C114-0A, Catachem, Oxford, CT, USA) and triglyceride standard (998-02992; Multi-calibrator lipids, Fujifilm Wako Diagnostics; Osaka, Japan) as reported previously [24]. Serum free fatty acid (FFA) concentrations were determined enzymatically with a modified plate assay, as previously described, using a serial dilution standard curve of FFA (276-76491; NEFA Standard Solution, Fujifilm Wako Diagnostics; Osaka, Japan) and VETSPEC reagents (C541-0A, Catachem; Oxford, CT, USA) [24]. The serum total protein (TP) concentration was determined using a Pierce BCA Protein Assay kit (23227; Thermo Fisher Scientific; Waltham, MA, USA) and a serially diluted bovine serum albumin standard provided in the assay. An internal control sample was included on each plate or autoanalyzer run, with an overall intra-assay CV of less than 10% and an inter-assay CV of less than 6.5% across all assays.

### 2.3. Statistical Analysis

Data were analyzed using repeated measures in SAS (version 9.4, SAS Inc., Cary, NC, USA), using the fixed effects of linear and quadratic dam BCS as continuous variables, as well as time, the interaction of time and BCS, and the random effect of calf. The day was specified in the repeated statement, and a first-order autoregressive structure for heterogenous variances was specified. Quadratic effects were removed from the model when *p* > 0.10. While not of primary interest, the fixed effect of dam dietary treatment was tested and subsequently removed when *p* > 0.10. Data were analyzed separately for each breed because calves of each breed were fed and housed differently.

Model assumptions were evaluated using externally studentized residuals. To meet model expectations, dependent variable transformations were required. Estimated least square means and the corresponding 95% confidence intervals were presented. Pairwise comparisons for differences between days were conducted using a Tukey–Kramer adjustment in order to avoid the inflation of a Type I error rate due to multiple comparisons, and a Bonferroni adjustment was used to separate the means for interactions.

## 3. Results and Discussion

Dairy cows present a unique model with which to determine the gestational effect on offspring performance given that calves are routinely separated from the dam at birth and are offered a consistent and similar diet across animals. There is a growing body of evidence that maternal factors, such as cow cooling, diet, and BCS, can have both short- and long-term effects on offspring performance [25,26,27]. Of particular interest in this study is cow BCS, an indicator of the body energy status over a period of time and an important management indicator [7]. Several studies have assessed the impact of dam BCS on offspring, demonstrating both immediate effects on neonatal calf growth and metabolism, and long-term production outcomes [8,9]. Considering that the LS may present a mechanistic mode of action for long-term feed intake regulation and metabolic control, the influence of dam BCS on the dairy calf neonatal leptin, cortisol, and metabolic profile could be useful in explaining the previously observed gestational effects on subsequent performance.

### 3.1. Leptin

**Figure 1:**
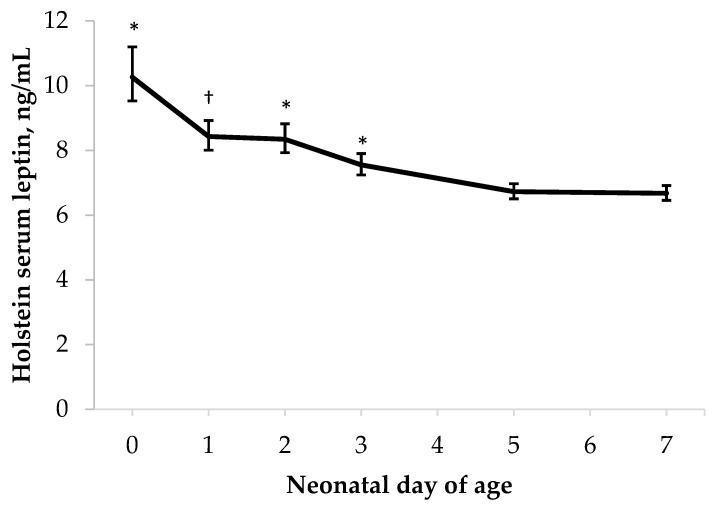
Effect of time on neonatal Holstein calf serum leptin concentration (*p* = 0.09). Means are reported with 95% confidence intervals. [* Denotes day is different than all subsequent days; † Denotes day is different from day 0, 3, 5, and 7].

There was no evidence of an association between BCS and leptin concentration in HOL or HOL-ANG calves (*p* ≥ 0.12; Table 1). Time tended to have an effect on the HOL leptin concentration (*p* = 0.09; Table 1) whereby leptin consistently declined from birth through day 5 (Figure 1). A decline in the circulating leptin concentration after birth was also previously observed in Holstein calves by Schäff et al. [28], but many researchers have observed a peak in neonatal leptin concentration in dairy calves at 2 d of age [14,29,30]. The circulating leptin concentration in suckling neonatal dairy calves [31] and beef calves [15,32,33] increases through the first few days of life, but remains elevated or exhibits a less marked decline over the following 2 weeks. Additional sampling beyond the 7 d window examined in the present study may have yielded additional useful information.

It is unclear why the leptin concentration in this study tended to decline in the HOL calves from birth, along with a numerical decline in HOL-ANG calves. In our study, calves were fed colostrum once at birth and milk replacer thereafter. Although a study with a similar feeding regimen resulted in a LS [34], most of the studies reporting a successful LS fed the calves with colostrum or transition milk for at least 3 d [14,30] or allowed the calf to suckle [15,32,33]. One potential explanation for the lack of LS in the current study is that bioactive components in whole milk or colostrum may increase circulating leptin [35], or that the colostrum may have a greater energy density than milk replacer (depending on replacer formulation) [36,37]. To assess the non-nutritive effects of colostrum vs. milk replacer, Liermann et al. [29] fed calves either colostrum or milk replacer formulated for a similar nutrient profile for the first 2 d of life. The colostrum-fed calves had a LS, but calves fed milk replacer had a declining leptin concentration similar to calves in the current study [29]. Schäff et al. [28] observed a leptin decline in calves fed either colostrum or milk replacer in another study. Additionally, long-chain fatty acids differently reduce the expression of leptin mRNA in cultured bovine adipocytes [38], and an increasing FA chain length over 8 C drastically inhibits leptin secretion in cultured rat adipocytes [39]. Therefore, it may be possible that the FA profile of the milk replacer used in the study herein could have influenced the circulating leptin concentration in the calves. In general, it seems that the bioactive components or energy density of the colostrum and transition milk may be beneficial factors for the LS, and early life nutrition should be considered when evaluating the LS in dairy calves in the future.

### 3.2. Cortisol

**Figure 2:**
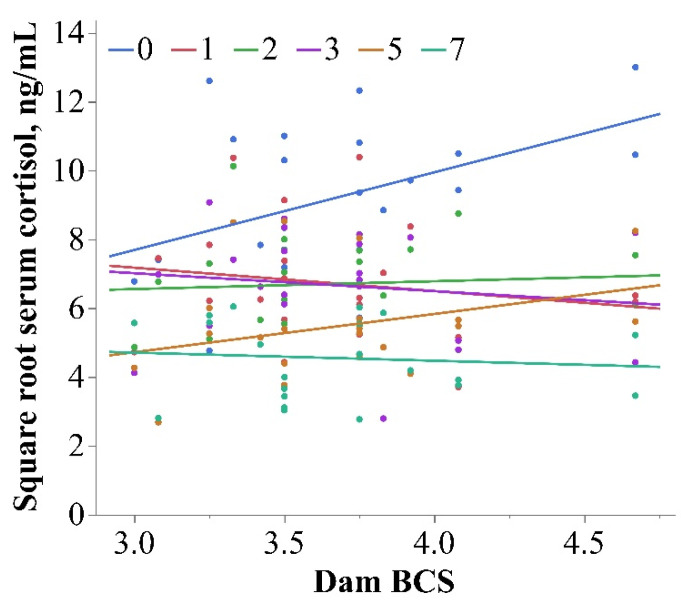
Interaction between dam BCS and time for neonatal serum cortisol in Holstein calves (*p* = 0.02; square root transformed). The d 0 serum cortisol was positively associated with dam BCS (blue line; *p* = 0.01; R^2^ = 0.20).

There was an interaction between BCS and time on the HOL cortisol concentration (*p* = 0.02; Table 1), and the daily cortisol mean concentrations are reported in Table 2. Th neonatal serum cortisol concentration and dam BCS were positively associated on day 0 (*p* = 0.01; Figure 2), but there was no evidence of an association on the remaining days of neonatal age (*p* ≥ 0.18). Other researchers have reported elevated cortisol in the first days of life for offspring of overweight dams. In lambs born to obese ewes, the plasma cortisol concentration was greater for the first 2 d of life compared with lambs from ewes with normal weight at parturition [13], and this effect was carried over to the second generation offspring [19].

In adult animals, cortisol increases leptin mRNA expression and secretion [40], including in bovine adipose tissue [41]. However, this relationship appears to differ in the immediate neonatal period as cortisol decreases while leptin concentration increases over the first week of life in sheep [42]. Research by Lewis et al. [32] demonstrated the potential direct role of glucocorticoids in the regulation of the neonatal LS, whereby the administration of hydrocortisol sodium succinate to calves at birth and 24 h of age eliminated the LS in beef calves [32]. In sheep, the administration of glucocorticoids to the dam during gestation had similar negative effects on the LS [43]. It has been hypothesized that the elevated glucocorticoid concentrations noted in the offspring of obese dams at birth may not exhibit the characteristic positive relationship between cortisol and leptin because of immature signaling or the production in neonatal adipose tissue [13], although attempts to demonstrate this mechanistically in the neonate have not been conducted to our knowledge. As we proposed earlier, it is possible that the milk feeding strategy of only offering colostrum at one feeding in the current study prevented the normal neonatal LS in our calves. Since the HOL calves had an elevated cortisol concentration at birth, as expected for the offspring of overweight dams, it would have been interesting to observe the relationship between serum cortisol and leptin had they been fed transition milk beyond the single colostrum feeding. 

### 3.3. Other Serum Metabolites

**Figure 3:**
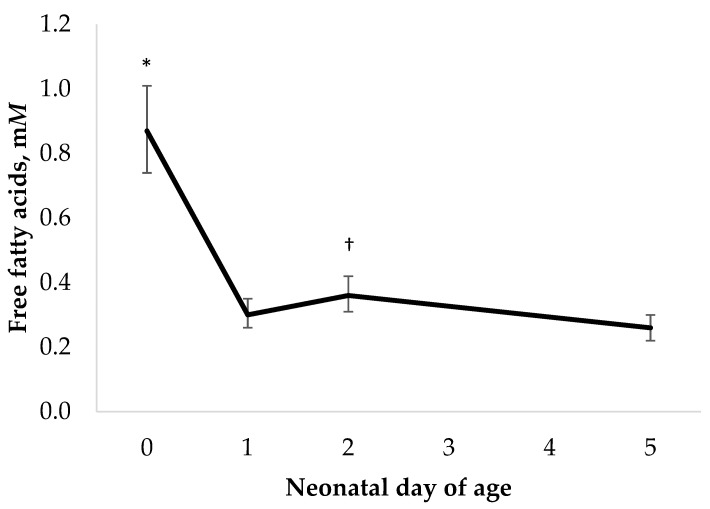
Effect of time on neonatal Holstein calf free fatty acid concentration (*p* = 0.01). Means are reported with 95% confidence intervals. [* Denotes that d 0 is different than all other days (*p* < 0.05); † Denotes a difference between day 2 and day 5 (*p* < 0.05)].

Dam BCS was positively associated with HOL calf BHB concentration (*p* < 0.01; Table 1), but not in HOL-ANG calves (*p* = 0.31; Table 1). This is in contrast to the results of Alharthi et al. [8], where plasma BHB was lower on the day of birth in calves born to dams with a greater dam BCS. Furthermore, there was no evidence of an effect of time on the circulating BHB concentration in HOL or HOL-ANG calves (*p* ≥ 0.19; Table 1). Measurements of BHB over the first week of life in calves are limited, but Diesch and colleagues [44] observed a decline in the circulating BHB concentration during the first 4 d of life. In general, the circulating BHB concentration in calves is considered an indicator of rumen development and the initiation of ruminal fermentation with the increased consumption of starter feeds over the first 6 weeks of life [45,46], and these changes are most notably observed around weaning [47,48]. Starter intake in these calves during the first week of life was negligible (52 g/d), and there was no relationship between the average serum BHB and week 1 starter intake (R^2^ < 0.02). It is unlikely that the changes we observed in the circulating BHB with the increasing dam BCS were related to starter intake. Another explanation for the greater BHB concentration with the increasing dam BCS in HOL calves may be the greater body fat mobilization and incomplete hepatic oxidation of FFA; however, there was no relationship between dam BCS and serum FFA for HOL or HOL-ANG calves (*p* ≥ 0.43). In fact, serum FFA decreased from the d 0 concentrations in both HOL and HOL-ANG calves (*p* = 0.01; Figure 3), which has also been observed in other studies [49]. Additional work is needed to understand the relationship between dam BCS and calf BHB concentration in the early neonatal period, as circulating BHB may be influenced by ruminal development or hepatic ketogenesis.

**Figure 4:**
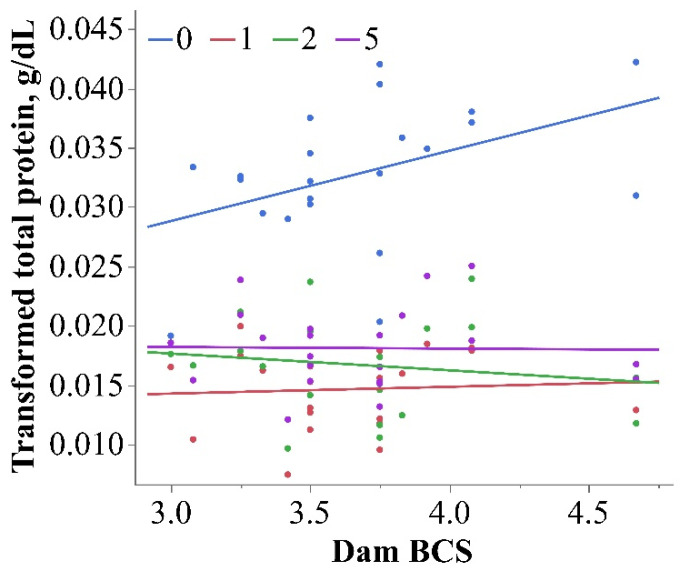
Interaction of dam BCS and time for neonatal serum total protein concentration in Holstein calves (*p* = 0.03; transformed 1/x^2^). The day 0 serum total protein was negatively associated with dam BCS (blue line; *p* < 0.01; R^2^ = 0.19).

The circulating TP concentration in neonatal calves is one indicator of the passive transfer of colostrum immunoglobulins. The interaction between dam BCS and TP (*p* = 0.03; Table 1) revealed a negative association between both variables in HOL calves on day 0 (*p* < 0.01), but not on other days (Figure 4). The overall means and 95% confidence intervals by day for TP are reported in Table 2. Albumin represents approximately half of the serum TP in the neonatal calf [50], and a high maternal BCS decreased the circulating calf albumin concentration at birth in a recent study [8]. In contrast, increasing the dam dietary energy density in the prepartum period does not affect the calf TP concentration [34]. The circulating TP concentration in neonatal calves is used as an indicator of the passive transfer of immunoglobulins; however, at the time of sampling on day 0 in the current study (within 4 h of birth), colostrum would not have been a factor in TP concentration because it can take up to 24 h for TP to increase following the first feeding [29]. Contrary to our results for HOL calves, the HOL-ANG calf TP concentration was positively associated with dam BCS (*p* = 0.01; Table 1), but there was no effect of time. The potential influence of the dam BCS on neonate TP concentrations at birth and throughout the first week of life warrants further investigation.

Despite the association between dam BCS and offspring serum TP, there was no evidence of an association between BCS and BUN (*p* ≥ 0.66; Table 1), suggesting that dam BCS does not influence protein metabolism. In contrast, calves born to dams with high BCS had depressed urea concentration at birth [8], with the authors speculating that there was greater amino acid deamination for use in gluconeogenesis. While the range in the BCS between our data and that of Alharthi et al. [8] is similar, there could be a myriad of factors beyond dam BCS that could create the observed discrepancy for offspring BUN, such as dam nutritional factors or calf rearing protocols. Furthermore, in the current study, there was no evidence of a change in serum BUN over time in either breed (*p* ≥ 0.39; Table 1), which aligns with another study in which calves were fed colostrum and transition milk for 3 d [49]. However, other researchers observed a marked increase in BUN concentration through day 3 of age in mixed dairy breed male calves when fed the first colostrum from powder for 3 d [49,51]. This contrasts with our study, where calves were fed the first colostrum at the initial feeding and may be a reason for the discrepancy between datasets. Overall, the inconsistency in the literature and dearth of data available regarding neonatal BUN indicates that more research is needed to fully understand the factors contributing to protein metabolism in the neonatal dairy calf under 1 week of age.

## 4. Conclusions

Dam BCS was not associated with offspring leptin concentration, and the serum leptin unexpectedly declined with age. The lack of LS may have been reflective of the milk replacer feeding program, although this study was not designed to directly compare different milk feeding strategies. Future studies should consider early life colostrum and milk feeding strategies when evaluating the bovine LS. In HOL, the dam BCS was associated with an increase in the offspring serum cortisol concentration, as expected, and supports previous work highlighting the potential stress imparted on the offspring by an excessive dam body condition during gestation. The dam BCS altered factors related to fatty acid metabolism and the serum protein concentration, but the mechanisms of action for those changes are not clear. Overall, elevated dam BCS at birth appears to alter the offspring metabolic profile. Controlled studies administering moderate or excessive gestational energy intake may assist in elucidating the impacts of the maternal plane of nutrition on neonatal calf metabolism, and further work is necessary in order to understand how these gestational modifications may influence the long-term performance in dairy cattle.

## Figures and Tables

**Table 1:** Parameter estimate and the statistical significance of the association among dam BCS, the neonatal day of age and serum concentration of leptin, cortisol and other blood metabolites in Holstein and Angus-sired calves ^1^.

	Dam BCS ^2^		*p*-Value			
Item	BCS Estimate	95% CI	BCS	Time	BCS × Time	Diet
Holstein, n = 22						
Leptin, ng/mL ^3^	−0.003	[−0.007, 0.001]	0.12	0.09	0.79	-
Cortisol, ng/mL ^4^	−0.24	[−1.87, 1.40]	0.43	0.08	0.02	-
BHB, mM ^5^	0.87	[0.31, 1.43]	<0.01	0.19	0.28	0.06
BUN, mg/dL ^4^	−0.30	[−0.83, 0.23]	0.66	0.46	0.32	-
FFA, mM ^4^	0.08	[−0.28, 0.44]	0.45	0.01	0.26	-
TG, mg/dL ^6^	0.005	[−0.004, 0.013]	0.24	0.26	0.39	-
Total Protein, g/dL ^3^	0.00	[−0.004, 0.004]	0.32	0.64	0.03	-
Holstein × Angus, n = 11						
Leptin, ng/mL ^3^	−0.006	[−0.018, 0.007]	0.68	0.51	0.58	-
Cortisol, ng/mL ^4^	−1.73	[−4.96, 1.49]	0.13	0.88	0.91	-
BHB, mM	0.02	[−0.09, 0.12]	0.31	0.53	0.58	0.06
BUN, mg/dL	0.65	[−0.85, 2.16]	0.98	0.39	0.41	-
FFA, mM ^4^	−0.01	[−0.34, 0.32]	0.43	0.27	0.49	-
TG, mg/dL ^3^	−0.001	[−0.003, 0.001]	0.15	0.86	0.91	-
Total Protein, g/dL	2.35	[0.47, 4.23]	0.01	0.45	0.20	0.02

^1^ Back-transformed data by day and breed that are not presented in the following tables and figures can be found in the supplemental file for informational purposes. ^2^ Estimates and confidence intervals are reported for the transformed data according to the following transformations: ^3^ (1/(x^2^)); ^4^ square root; ^5^ natural log; ^6^ 1/(x).

**Table 2:** Least square means and 95% confidence interval of serum cortisol and total protein by day of age in neonatal Holstein heifer calves.

Day	Cortisol, mg/dL		Total Protein, g/dL	
0	84.7	[72.5, 97.9]	5.5	[5.3, 5.6]
1	45.0	[36.3, 54.8]	8.3	[7.8, 8.8]
2	44.9	[36.1, 54.6]	7.7	[7.4, 8.2]
3	44.3	[35.6, 54.0]	-	
5	29.8	[22.8, 37.9]	7.7	[7.4, 8.2]
7	20.6	[14.8, 27.4]	-	

## Data Availability

Data will be made available upon request to the corresponding author due to privacy.

## Supplementary Materials

The following supporting information can be downloaded at: https://www.mdpi.com/article/10.3390/metabo13050631/s1, Table S1: Least square means and 95% confidence intervals of blood metabolites in female Holstein calves by neonatal day of age; Table S2: Least square means and 95% confidence intervals of blood metabolites in female Angus × Holstein calves by neonatal day of age.
