# Supplementary material for: Dam Body Condition Score Alters Offspring Circulating Cortisol and Energy Metabolites in Holstein Calves but Did Not Affect Neonatal Leptin Surge
Source: Metabolites. 2023 May 6;13(5):631. doi: 10.3390/metabo13050631 (PMC10221655; doi:10.3390/metabo13050631)
Supplement: Supplementary file 1 [file metabolites-13-00631-s001.zip › metabolites-2344366-supplementary.pdf]

Supplemental Table S1. Least square means and 95% confidence intervals of blood metabolites in female Holstein calves by neonatal day of age.

Day	β-hydroxybutyrate, m*M*		Blood urea nitrogen, mg/dL		Triglyceride, mg/dL	
0	0.083	[0.066, 0.116]	11.8	[10.3, 13.3]	27.4	[24.9, 30.4]
1	0.091	[0.072, 0.115]	12.0	[10.5, 13.6]	23.2	[21.3, 25.3]
2	0.110	[0.086, 0.140]	10.2	[8.8, 11.7]	26.9	[24.6, 22.6]
5	0.071	[0.056, 0.090]	9.1	[7.8, 10.5]	20.9	[19.5, 22.6]

Supplemental Table S2. Least square means and 95% confidence intervals of blood metabolites in female Angus x Holstein calves by neonatal day of age.

Day	β-hydroxybutyrate, m*M*	Urea nitrogen, mg/dL	Cortisol, mg/dL	Free fatty acids, m*M*	Leptin, ng/mL	Triglyceride, mg/dL	Total protein, g/dL
0	0.108 [0.085, 0.131]	9.3 [7.3, 11.5]	66.3 [54.7, 79.1]	0.90 [0.76, 1.05]	9.2 [8.2, 10.6]	29.9 [25.3, 38.4]	5.1 [4.8, 5.5]
1	0.075 [0.053, 0.098]	8.0 [6.2, 10.1]	23.5 [16.9, 31.3]	0.33 [0.24, 0.42]	7.3 [6.8, 8.0]	26.1 [31.2, 22.9]	8.3 [7.9, 8.6]
2	0.062 [0.040, 0.084]	6.8 [5.1, 8.8]	27.4 [20.2, 35.8]	0.28 [0.20, 0.36]	7.1 [6.6, 7.6]	26.5 [23.2, 31.9]	8.0 [7.6, 8.3]
3	-	-	20.5 [14.3, 27.8]	-	6.9 [6.5, 7.4]	-	-
5	0.053 [0.030, 0.076]	7.6 [5.8, 9.6]	19.3 [13.3, 26.4]	0.28 [0.21, 0.37]	6.5 [6.1, 6.8]	21.3 [19.4, 23.9]	7.4 [7.1, 8.0]
7	-	-	14.4 [9.3, 20.6]	-	6.4 [6.1, 6.8]	-	-
